# Risk of tuberculosis disease in patients receiving TNF-α antagonist therapy: A meta-analysis of randomized controlled trials

**DOI:** 10.1016/j.nmni.2024.101533

**Published:** 2024-11-16

**Authors:** Fatemeh Khelghati, Mohammad Rahmanian, Elaheh Eghbal, Zahra Sadat Seghatoleslami, Mehdi Goudarzi, Aliasghar Keramatinia, Catherine WM. Ong, Delia Goletti, Lia D'Ambrosio, Rosella Centis, Mohammad Javad Nasiri, Giovanni Battista Migliori

**Affiliations:** a Department of Microbiology, School of Medicine, Shahid Beheshti University of Medical Sciences, Tehran, Iran; b Infectious Diseases and Tropical Medicine Research Center, Shahid Beheshti University of Medical Sciences, Tehran, Iran; c Department of Infectious Diseases, Islamic Azad University, Tehran Medical Branch, Tehran, Iran; d Department of Community Medicine, School of Medicine, Shahid Beheshti University of Medical Sciences, Tehran, Iran; e Division of Infectious Diseases, Department of Medicine, National University Hospital, Singapore, Singapore; f Translational Research Unit, Department of Epidemiology and Preclinical Research National Institute for Infectious Diseases L. Spallanzani-IRCCS, Roma, Italy; g Public Health Consulting Group, Lugano, Switzerland; h Servizio di Epidemiologia Clinica delle Malattie Respiratorie, Istituti Clinici Scientifici Maugeri IRCCS, Tradate, Italy

**Keywords:** Tuberculosis, TNF-α antagonist therapy, Autoimmune diseases, Rheumatoid arthritis, Adalimumab, Golimumab, Infliximab, Certolizumab pegol, Etanercept

## Abstract

**Introduction:**

Tuberculosis (TB) risk associated with tumor necrosis factor-alpha (TNF-α) antagonist therapy in patients with autoimmune diseases is a significant concern. This study aims to evaluate the risk of TB disease associated with TNF-α antagonist therapy.

**Methods:**

An extensive search of PubMed/MEDLINE, EMBASE, and the Cochrane CENTRAL databases was conducted to identify randomized controlled trials (RCTs) assessing TB disease risk in patients receiving TNF-α antagonist therapy available until November 1, 2024. The pooled statistic used was the weighted odds ratio (OR) and a corresponding 95 % confidence interval (CI). Statistical analysis was performed using Comprehensive Meta-Analysis software, version 3.0 (Biostat Inc., Englewood, NJ, USA).

**Results:**

Fifty-six RCTs, totaling 22,212 adult patients, met the specified eligibility criteria. Pooled analysis revealed an increased risk of TB disease associated with TNF-α antagonist therapy (OR 1.52, 95 % CI 1.03–2.26, p = 0.03). Subgroup analyses indicated a higher risk in patients with rheumatoid arthritis (RA) (OR 2.25, 95 % CI 1.13–4.45, p = 0.02), while no significant associations were found in patients with ankylosing spondylitis (AS) or psoriasis (Ps). Analyses by specific TNF-α antagonist drugs did not yield significant associations with risk of TB disease.

**Conclusion:**

Our study highlights an increased risk of TB disease associated with TNF-α antagonist therapy, particularly in patients with RA. However, the absence of significant associations in AS or Ps patients suggests disease-specific variations in risk of TB disease. Further research is needed to elucidate the long-term safety profile of TNF-α antagonist drugs and their associations with risk of TB disease in different patient populations.

## 1 Introduction

Tuberculosis (TB) remains a significant global health concern, with approximately 10 million new cases and 1.4 million deaths reported worldwide each year [1,2]. Among individuals receiving immunosuppressive therapy for autoimmune diseases, such as rheumatoid arthritis (RA), ankylosing spondylitis (AS), psoriasis (Ps), Crohn's Disease (CD), and Ulcerative Colitis (UC), the risk of TB reactivation is of particular concern [[3], [4], [5], [6], [7]]. Tumor necrosis factor-alpha (TNF-α) antagonists, a class of biologic agents, have revolutionized the treatment of autoimmune diseases by targeting the inflammatory cascade [[8], [9], [10]]. However, TNF-α plays a crucial role in host defense against TB, and its inhibition may predispose patients to TB infection or reactivation [8,11,12].

While the association between TNF-α antagonists and risk of TB disease has been studied, a comprehensive and updated analysis has not yet been performed.

In light of these considerations, our systematic review and meta-analysis aim to comprehensively evaluate the risk of TB disease associated with TNF-α antagonist therapy across various autoimmune diseases and specific TNF-α antagonist drugs.

## 2 Methods

### 2.1 Search strategy

We performed an extensive search of PubMed/MEDLINE, EMBASE, and the Cochrane CENTRAL databases to identify relevant studies available until November 1, 2024, This study adhered to PRISMA statement for its design and reporting (Prospero ID: CRD42024513989) [13]. The following key terms were utilized in combination with mesh terms: ‘Tuberculosis’, 'TNF-α antagonist', 'etanercept', 'adalimumab', 'infliximab', 'golimumab', and 'certolizumab'.

### 2.2 Study selection

The collected records from the database searches were merged, and duplicates were removed through the utilization of EndNote X7 (Thomson Reuters, Toronto, ON, Canada). Study selection was performed in two steps: a thorough assessment of records was conducted individually, using title/abstract and full-text screening to exclude any studies that did not align with the study's objectives.

The randomized controlled trials (RCTs) included in the analysis met the following criteria based on the Population, Intervention, Comparison, and Outcomes (PICO):

*Participants:* Adult patients with a range of autoimmune diseases, including but not limited to RA, Ps, and AS, treated with TNF-α antagonists.

*Interventions:* TNF-α antagonists Golimumab (GOL), Adalimumab (ADA), Certolizumab (CZP), Infliximab (IFX), and Etanercept (ETN) with or without standard-care treatment for any medical condition.

*Comparison:* Placebo with or without standard-care treatment.

*Outcome:* TB risk in patients treated with TNF-α antagonists.

We specifically included RCTs to ensure a high level of evidence. The primary outcome assessed in the included studies was an improvement in rheumatological signs.

### 2.3 Data extraction

A structured data extraction form was employed to gather data from all qualifying studies. Discrepancies were addressed through mutual agreement. The extracted data encompassed several aspects, including the primary author's name, publication year, study duration, study type, demographic details, geographical location(s) of the study, sample size, interventions/comparisons and outcomes.

### 2.4 Quality assessment

Quality assessment of the included studies was performed and peer-reviewed to avoid discrepancies, using the Cochrane tool [14]. This assessment tool encompasses several domains, encompassing random sequence generation, allocation concealment, blinding of participants and personnel, blinding of outcome assessors, completeness of outcome data, as well as additional factors like selective reporting and potential biases. Each individual study underwent categorization based on bias risk: low risk of bias when no bias concerns were detected, high risk of bias when bias concerns were present, or unclear risk of bias when there was an insufficient amount of information available for evaluation.

### 2.5 Data analysis

The statistical analysis was performed using Comprehensive Meta-Analysis software, version 3.0 (Biostat Inc., Englewood, NJ, USA). Weighted odd ratio (OR) was used as the pooled statistic, with a corresponding 95 % confidence interval (CI). In cases where the statistical heterogeneity between the studies was low (I^2^ ≤ 50 % or p ≥ 0.1), the fixed-effect model was utilized. Conversely, if a significant level of inter-study heterogeneity was observed (I^2^ > 50 % or p < 0.1), the random-effects model was employed. Cochran's Q test and the I^2^ statistic were used to assess between-study heterogeneity. To evaluate publication bias, Begg's test was applied, where a P value of <0.05 was considered indicative of statistically significant publication bias.

## 3 Results

**Fig. 1:**
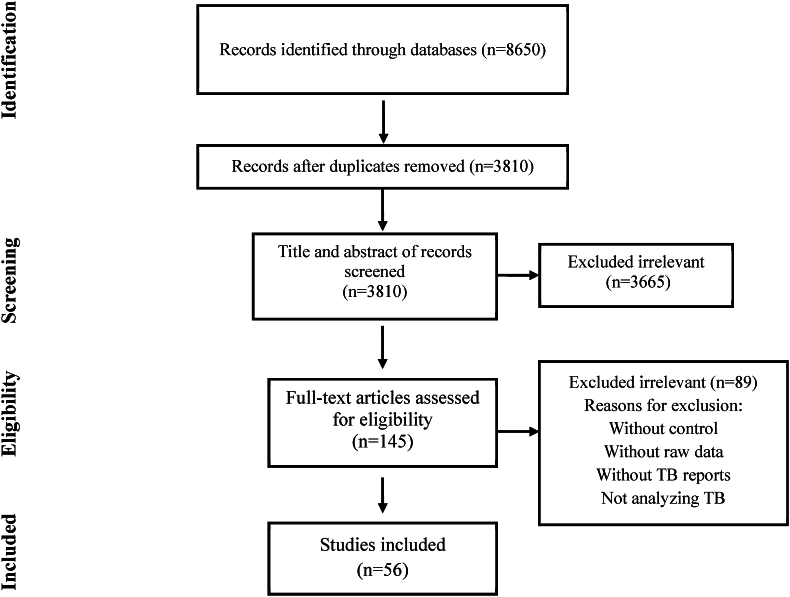
Flow chart of study selection for inclusion in the systematic review and meta-analysis.

Fig. 1 illustrates the flow diagram of the systematic review process. This thorough review yielded a total of 56 records that met the specified eligibility criteria [[15], [16], [17], [18], [19], [20], [21], [22], [23], [24], [25], [26], [27], [28], [29], [30], [31], [32], [33], [34], [35], [36], [37], [38], [39], [40], [41], [42], [43], [44], [45], [46], [47], [48], [49], [50], [51], [52], [53], [54], [55], [56], [57], [58], [59], [60], [61], [62], [63], [64], [65], [66], [67], [68], [69], [70]]. The included studies encompassed a total of 13,913 adult patients who received TNF-α antagonists and 8299 received a placebo (Table 1). In the intervention group, raw data for IGRA were reported in only 10 studies, involving 1443 patients with negative IGRA, while others used tuberculin skin tests, chest radiography, or clinical evaluations. Additionally, steroid use in the intervention group was documented in 33 studies, with a total of 4030 patients receiving steroid treatment. These trials targeted a range of diseases, including AS, RA, Ps, CD, UC, and others. The average time of follow-up since randomization across the included studies was approximately 38 weeks (range 4 weeks–256 weeks).

**Table 1:** Characteristics of included studies.

First Author	Year	Country	Design of study	Disease	Time of follow-up since randomization (Weeks)	Intervention (TNF-α antagonist)	Comparison
Deodhar [28]	2022	Multicenter	Phase 3 RCT	AS	52	GOL	PBO
Combe [26]	2021	Multicenter	Phase 3 RCT	RA	54	ADA + MTX	PBO + MTX
Umezawa [65]	2021	Japan	Phase 3 RCT	Ps	52	CZP	PBO
Baili Chen [23]	2020	Multicenter	Phase 3 RCT	CD	4	ADA	PBO
Pavelka [48]	2020	Multicenter	Phase 3 RCT	RA	26	ADA + MTX	PBO + MTX
Fleischmann [30]	2019	USA	Phase 3 RCT	RA	26	ADA + MTX	PBO + MTX
Elewski [29]	2018	Multicenter	Phase 3 RCT	Ps	26	ADA	PBO
Kang [35]	2018	Korea	Phase 3 RCT	RA	24	CZP + MTX	PBO + MTX
Lebwohl-1 [43]	2018	Multicenter	Phase 3 RCT	Ps	12	CZP	PBO
Lebwohl-2 [43]	2018	Multicenter	Phase 3 RCT	Ps	12	ETN	PBO
Reinisch [50]	2018	USA	Phase 3 RCT	UC	54	GOL	PBO
Cai [22]	2017	China	Phase 3 RCT	Ps	12	ADA	PBO
Mease [46]	2017	USA	Phase 3 RCT	Ps	24	ADA	PBO
Kavanaugh-1 [37]	2017	USA	Phase 3 RCT	PsA	24	GOL	PBO
Reveille [51]	2017	Multicenter	Phase 3 RCT	AS	16	GOL	PBO
Taylor-1 [64]	2017	Multicenter	Phase 3 RCT	RA	24	ADA	PBO
Armstrong [15]	2016	USA and Canada	Phase 3 RCT	Ps	16	ADA	PBO
Atsumi [16]	2016	Japan	Phase 3 RCT	RA	52	CZP + MTX	PBO + MTX
Jaffe [33]	2016	Multicenter	Phase 3 RCT	Active Noninfectious Uveitis	80	ADA	PBO
Keystone-1 [40]	2016	USA	Phase 3 RCT	RA	256	GOL + MTX	PBO + MTX
Maksymowych [45]	2016	Multicenter	Phase 3 RCT	SpA	12	ETN	PBO
Bingham [19]	2015	Multicenter	Phase 3 RCT	RA	16	GOL + MTX	PBO + MTX
Gordon [31]	2015	Multicenter	Phase 2 RCT	Ps	16	ADA	PBO
Rutgeerts-1 [52]	2015	Multicenter	Phase 2 RCT	UC	6	GOL	PBO
Sieper-1 [58]	2015	Multicenter	Phase 3 RCT	SpA	16	GOL	PBO
Taylor-2 [63]	2015	Multicenter	Phase 2 RCT	RA	6	ADA	PBO
Judson [34]	2014	USA	Phase 2 RCT	Sarcoidosis	44	GOL	PBO
Kennedy [38]	2014	Multicenter	Phase 2 RCT	RA	12	ADA	PBO
Nam [47]	2014	Multicenter	Phase 4 RCT	RA	78	IFX + MTX	PBO + MTX
Schiff-1 [56]	2014	Multicenter	Phase 3 RCT	RA	96	ADA + MTX	ABA + MTX
Sieper-2 [57]	2014	Multicenter	Phase 3 RCT	AS	28	IFX + NPX	PBO + NPX
Suzuki [61]	2014	Japan	Phase 2/3	UC	8	ADA	PBO
Huang [32]	2014	China	Phase 3 RCT	AS	12	ADA	PBO
Kavanaugh-2 [36]	2013	Multicenter	Phase 4 RCT	RA	26	ADA + MTX	PBO + MTX
Sandborn [54]	2013	Multicenter	Phase 3 RCT	UC	54	GOL	PBO
Baranauskaite [17]	2012	USA	Phase 3 RCT	PsA	16	IFX + MTX	MTX
Reich [49]	2012	Multicenter	Phase 2 RCT	Ps	12	CZP	PBO
Tam [62]	2012	China	Phase 3 RCT	RA	72	IFX + MTX	MTX
Barker [18]	2011	Multicenter	Phase 3 RCT	Ps	24	IFX	MTX
van Vollenhoven [68]	2011	Multicenter	Phase 2 RCT	RA	24	ADA + MTX	PBO + MTX
Colombel [25]	2010	Multicenter	Phase 3 RCT	CD	20	IFX/IFX + AZA	AZA
Chen [24]	2009	USA	Phase 3 RCT	RA	12	ADA + MTX	MTX
Couriel [27]	2009	USA	Phase 3 RCT	GvH	6	IFX + MP	MP
Smolen [59]	2009	Multicenter	Phase 3 RCT	RA	24	CZP + MTX	PBO + MTX
Wenzel [69]	2009	Multicenter	Phase 2 RCT	Asthma	76	GOL	PBO
Keystone-2 [39]	2008	USA	Phase 3 RCT	RA	52	CZP + MTX	PBO + MTX
Schiff-2 [55]	2008	Multicenter	Phase 3 RCT	RA	48	IFX + MTX	PBO + MTX
Kim [42]	2007	Korea	Phase 3 RCT	RA	24	ADA	PBO
Heijde [67]	2007	Multicenter	Phase 3 RCT	RA	96	ETN/ETN + MTX	MTX
Breedveld [21]	2006	Multicenter	Phase 3 RCT	RA	96	ADA/ADA + MTX	MTX
Westhovens [70]	2006	Multicenter	Phase 3 RCT	RA	22	IFX + MTX	PBO + MTX
Rutgeerts-2 [53]	2005	Multicenter	Phase 3 RCT	UC	54	IFX	PBO
Keystone [41]	2004	USA	Phase 3 RCT	RA	52	ADA + MTX	PBO + MTX
Clair [60]	2004	USA	Phase 3 RCT	RA	54	IFX + MTX	PBO + MTX
Braun [20]	2002	Multicenter	Phase 3 RCT	AS	12	IFX	PBO
Bosch [66]	2002	Belgium	Phase 3 RCT	AS	12	IFX	PBO
Maini [44]	1999	Multicenter	Phase 3 RCT	RA	102	IFX + DMARDs	DMARDs

AS (Ankylosing Spondylitis), RA (Rheumatoid Arthritis), Ps (Psoriasis), CD (Crohn's Disease), UC (Ulcerative Colitis), PsA (Psoriatic Arthritis), SpA (Spondyloarthritis), GvH (Graft versus Host Disease). Golimumab (GOL), Adalimumab (ADA), Certolizumab Pegol (CZP), Etanercept (ETN), and Infliximab (IFX) were used alongside placebo (PBO), methotrexate (MTX), and other medications such as Abatacept (ABA), Naproxen (NPX), Azathioprine (AZA), Mercaptopurine (MP), and Disease-modifying antirheumatic drugs (DMARDs).

The interventions consisted of TNF-α antagonists such as GOL, ADA, Certolizumab CZP, IFX, and ETN, administered either alone or in combination with other drugs like methotrexate (MTX). These interventions were compared against control groups, typically placebos or standard of care treatment for the respective rheumatological conditions.

### 3.1 Quality assessment

Across the board, all studies scored positively in terms of random sequence generation, allocation concealment, and blinding of participants and outcome assessors, with each receiving a "Yes" rating (Table 2). This indicates that the trials were conducted with proper randomization techniques, allocation concealment methods, and efforts to mitigate bias by blinding participants and assessors to treatment allocation. Additionally, none of the studies exhibited incomplete outcome data or selective reporting, with all receiving a "No" rating in these areas. Furthermore, there were no indications of other biases in the included studies. Overall, these findings suggest an acceptable level of methodological rigor and adherence to quality standards in the design and execution of the trials, bolstering the reliability of their outcomes and conclusions.

**Table 2:** Quality assessment.

First Author	Random Sequence Generation	Allocation Concealment	Blinding of Participants	Blinding of Outcome Assessors	Incomplete Outcome Data	Selective reporting	Other bias
Deodhar	Yes	Yes	Yes	Yes	No	No	No
Combe	Yes	Yes	Yes	Yes	No	No	No
Umezawa	Yes	Yes	Yes	Yes	No	No	No
Baili Chen	Yes	Yes	Yes	Yes	No	No	No
Pavelka	Yes	Yes	Yes	Yes	No	No	No
Fleischmann	Yes	Yes	Yes	Yes	No	No	No
Elewski	Yes	Yes	Yes	Yes	No	No	No
Kang	Yes	Yes	Yes	Yes	No	No	No
Lebwohl-1	Yes	Yes	Yes	Yes	No	No	No
Reinisch	Yes	Yes	Yes	Yes	No	No	No
Cai	Yes	Yes	Yes	Yes	No	No	No
Mease	Yes	Yes	Yes	Yes	No	No	No
Kavanaugh-1	Yes	Yes	Yes	Yes	No	No	No
Reveille	Yes	Yes	Yes	Yes	No	No	No
Taylor-1	Yes	Yes	Yes	Yes	No	No	No
Armstrong	Yes	Yes	Yes	Yes	No	No	No
Atsumi	Yes	Yes	Yes	Yes	No	No	No
Jaffe	Yes	Yes	Yes	Yes	No	No	No
Keystone-1	Yes	No	No	No	No	No	No
Maksymowych	Yes	Yes	Yes	Yes	No	No	No
Bingham	Yes	Yes	Yes	Yes	No	No	No
Gordon	Yes	Yes	Yes	Yes	No	No	No
Rutgeerts-1	Yes	Yes	Yes	Yes	No	No	No
Sieper-1	Yes	Yes	Yes	Yes	No	No	No
Taylor-2	Yes	Yes	Yes	Yes	No	No	No
Judson	Yes	Yes	Yes	Yes	No	No	No
Kennedy	Yes	Yes	Yes	Yes	No	No	No
Nam	Yes	Yes	Yes	Yes	No	No	No
Schiff-1	Yes	Yes	Yes	Yes	No	No	No
Sieper-2	Yes	Yes	Yes	Yes	No	No	No
Suzuki	Yes	Yes	Yes	Yes	No	No	No
Huang	Yes	Yes	Yes	Yes	No	No	No
Kavanaugh-2	Yes	Yes	Yes	Yes	No	No	No
Sandborn	Yes	Yes	Yes	Yes	No	No	No
Baranauskaite	Yes	No	No	No	No	No	No
Reich	Yes	Yes	Yes	Yes	No	No	No
Tam	Yes	No	No	No	No	No	No
Barker	Yes	No	No	No	No	No	No
van Vollenhoven	Yes	Yes	Yes	Yes	No	No	No
Colombel	Yes	Yes	Yes	Yes	No	No	No
Chen	Yes	Yes	Yes	Yes	No	No	No
Couriel	Yes	No	No	No	No	No	No
Smolen	Yes	Yes	Yes	Yes	No	No	No
Wenzel	Yes	Yes	Yes	Yes	No	No	No
Keystone-2	Yes	Yes	Yes	Yes	No	No	No
Schiff-2	Yes	Yes	Yes	Yes	No	No	No
Kim	Yes	Yes	Yes	Yes	No	No	No
Heijde	Yes	Yes	Yes	Yes	No	No	No
Breedveld	Yes	Yes	Yes	Yes	No	No	No
Westhovens	Yes	Yes	Yes	Yes	No	No	No
Rutgeerts-2	Yes	Yes	Yes	Yes	No	No	No
Keystone	Yes	Yes	Yes	Yes	No	No	No
Clair	Yes	Yes	Yes	Yes	No	No	No
Braun	Yes	No	No	No	No	No	No
Bosch	Yes	Yes	Yes	Yes	No	No	No
Maini	Yes	Yes	Yes	Yes	No	No	No

### 3.2 Risk of TB disease and TNF-α antagonists

**Fig. 2:**
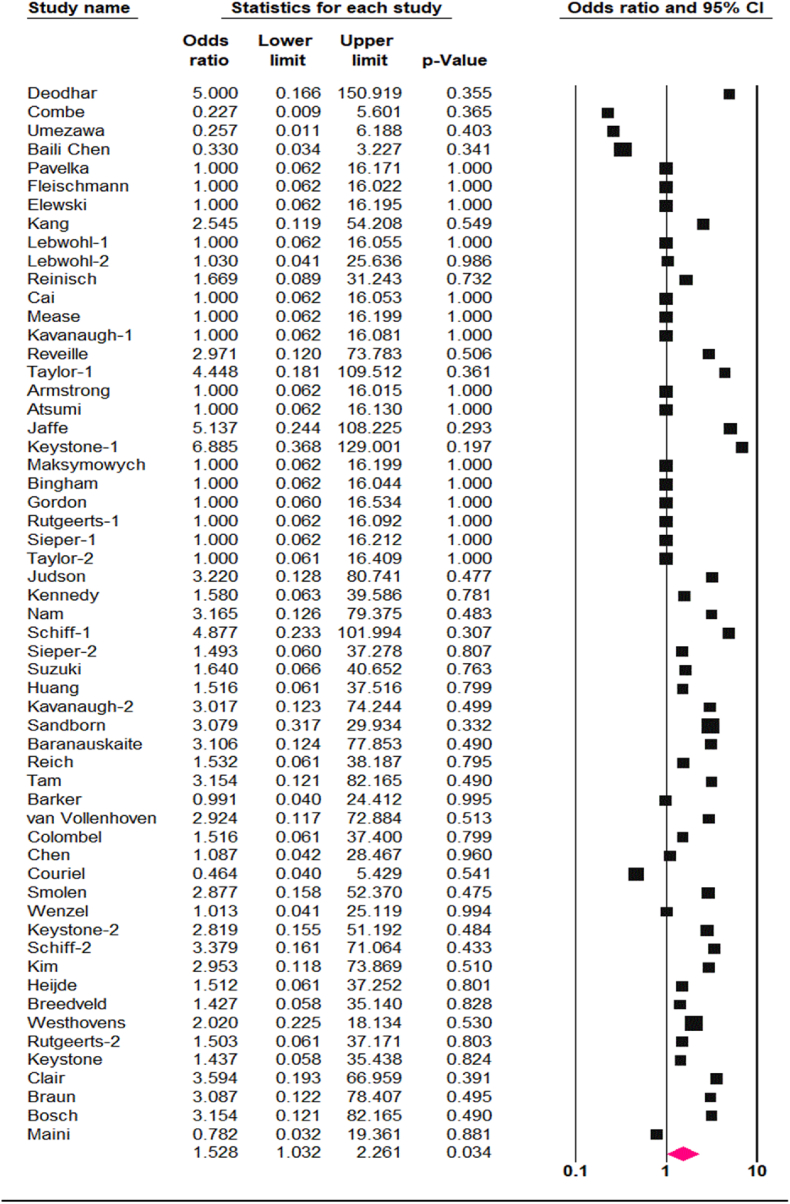
Pooled OR of TB disease risk associated with TNF-α antagonists.

**Fig. 3:**
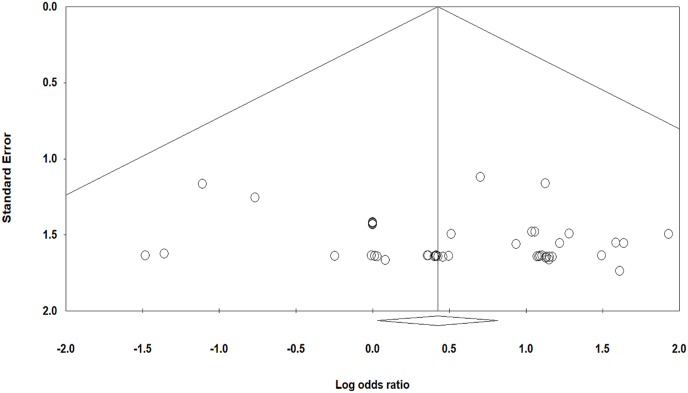
Funnel plot of included studies.

Among the 13,913 adult patients who received TNF-α antagonists, 68 developed TB disease comprising of 59 cases of pulmonary TB and 9 cases of disseminated TB. In the placebo arm of 8299 individuals, 8 developed TB disease. The pooled analysis determined that treatment with TNF-α antagonists was associated with an increased occurrence of TB disease compared with control groups (OR 1.52, CI95 % 1.03, 2.26, p = 0.03, I^2^ = 0 %) (Fig. 2). The funnel plot revealed no obvious asymmetry in distribution, suggesting a low likelihood of publication bias (Fig. 3), and this was statistically confirmed by Begg's test (p = 0.1) and Egger's regression asymmetry test (p = 0.2).

### 3.3 Subgroup analyses

The subgroup analysis delved into the impact of treatments and specific drugs across different diseases, shedding light on their associations with the risk of TB (Table 3). In terms of treatments, the analysis encompassed studies focusing on AS, Ps, and RA. While the pooled ORs for AS and Ps did not indicate significant associations, with values of 2.49 (95 % CI: 0.58–10.6) and 0.79 (95 % CI: 0.16–3.92) respectively, the results for RA revealed a notable association with an OR of 2.25 (95 % CI: 1.13–4.45) and a significant p-value of 0.02. However, heterogeneity was low across all analyses, and the Begg and Egger p-values were consistently above 0.5, suggesting minimal publication bias.

**Table 3:** Subgroup analysis.

Variables		No. of studies	No. of patients	Pooled OR	P value	I2%	Begg and Egger P value
**Disease-specific analyses**	**AS**	7	832	2.49 (0.58–10.6)	0.21	0.0	>0.5
	**Ps**	9	3680	0.79 (0.16–3.92)	0.77	0.0	>0.5
	**RA**	25	13083	2.25 (1.13–4.45)	0.02	0.0	>0.5
**Drug-specific analysis**	**ADA**	22	9784	1.56 (0.68–3.57)	0.28	0.0	>0.5
	**GOL**	11	3443	2.91 (0.95–8.91)	0.06	0.0	>0.5
	**IFX**	14	5168	1.82 (0.81–4.06)	0.14	0.0	>0.5
	**CZP**	8	2702	1.60 (0.41–6.25)	0.49	0.0	>0.5
	**ETN**	3	1115	1.24 (0.12–10.2)	0.19	0.0	>0.5

AS (Ankylosing Spondylitis), Ps (Psoriasis), RA (Rheumatoid Arthritis), ADA (Adalimumab), GOL (Golimumab), IFX (Infliximab), CZP (Certolizumab Pegol), ETN (Etanercept).

Further subgroup analysis focused on specific drugs within the TNF-α antagonist class, including ADA, GOL, IFX, CZP, and ETN. While none of the drugs exhibited a significant association with outcomes based on the pooled ORs and corresponding p-values, the analysis provided insights into their individual efficacies across different conditions. Notably, the examination of each drug's performance revealed no substantial heterogeneity and minimal evidence of publication bias, as indicated by consistent Begg and Egger p-values above 0.5.

## 4 Discussion

The study found that treatment with TNF-α antagonists increased the risk of TB disease compared to control groups (OR 1.52, CI95 % 1.03–2.26, p = 0.03), with subgroup analyses revealing a significant association with TB risk in RA patients (OR 2.25, CI95 % 1.13–4.45, p = 0.02), but not in AS or Ps patients. No specific TNF-α antagonist drugs showed significant associations with TB risk, suggesting that RA patients may face higher risk due to disease-specific immune factors and the use of additional immunosuppressive therapies like corticosteroids.

Comparing our findings with the study by Zhang et al. [71], our research offers a more comprehensive analysis, incorporating data from 56 RCTs to strengthen the evidence base (totaling 22,212 patients). This increase in sample size nearly doubles the statistical power, allowing us to assess TB risk across various conditions with greater precision. Additionally, our study includes RCTs published up to November 1, 2024, capturing more recent therapeutic data. While both studies conducted disease-specific analyses, our expanded dataset provides a clearer understanding of heightened TB risk in RA, as well as improved confidence intervals that narrow the uncertainty around TB risk levels associated with each TNF-α antagonist. Moreover, while both studies assessed individual TNF-α antagonists, our larger sample size allows for increased statistical confidence in drug-specific risk estimates.

Our findings carry important clinical implications. According to the World Health Organization (WHO) and local guidelines [72], TB screening is mandatory for patients undergoing TNF-α inhibitor therapy, with preventive treatment recommended for those diagnosed with latent TB infection [[73], [74], [75], [76]]. These procedures have proven effective, significantly decreasing TB incidence among rheumatological patients, as demonstrated in the UK BSRBR registry [77]. The observed association between TNF-α antagonists and increased TB risk highlights the necessity of TB screening and vigilant monitoring, especially for patients testing negative on IGRA or TST who may not receive preventive therapy [[78], [79], [80], [81]]. Subgroup analyses further emphasize the importance of personalized risk assessment based on specific rheumatological diseases. While TB risk exists for all patients treated with TNF-α antagonists, RA patients, in particular, may require enhanced monitoring due to their elevated risk profile compared to those with AS or Ps [[82], [83], [84]]. Clinicians should account for individual patient factors, disease severity, and treatment goals when initiating or continuing TNF-α antagonist therapy [[82], [83], [84]].

Our analysis of different TNF-α antagonist drugs did not reveal significant associations with TB risk for specific drugs, but it underscores the importance of considering the overall risk-benefit profile when selecting a TNF-α antagonist for a particular patient. Close monitoring for TB infection and appropriate preventive measures, including screening and preventive treatment where necessary, remain essential components of patient care [[85], [86], [87]].

Some limitations must be acknowledged. Our study was specifically designed to assess TB risk associated with TNF-α antagonist therapy, without evaluating a broad spectrum of adverse events. Future research could expand on these findings by exploring additional adverse events and long-term safety outcomes. Additionally, subgroup analyses by disease type and specific TNF-α antagonist were constrained by the availability of data within each subgroup, which may have influenced the robustness of our findings. Observational studies with longer follow-up periods may be required to thoroughly assess the long-term safety profile of TNF-α antagonists. Furthermore, the generalizability of our findings may be limited to the populations and settings represented in the included RCTs, as patients with specific comorbidities, co-medications, or demographic characteristics not well-represented in the trials may exhibit different risk profiles. Moreover, most studies in our analysis were multicenter, without stratification by country of origin, limiting our ability to assess whether regional TB burden influenced outcomes.

## 5 Conclusions

In Conclusion, our study underscores the importance of screening for TB infection in patients undergoing TNF-α antagonist therapy, particularly those with RA. While we found an increased TB disease risk associated with TNF-α antagonists, especially in RA patients, clinicians should carefully weigh the benefits and risks of treatment. Vigilance for TB infection and consideration of other potential adverse events are essential in clinical decision-making for patients receiving TNF-α antagonist therapy.

## CRediT authorship contribution statement

**Fatemeh Khelghati:** Methodology, Investigation. **Mohammad Rahmanian:** Methodology, Investigation. **Elaheh Eghbal:** Investigation, Conceptualization. **Zahra Sadat Seghatoleslami:** Investigation. **Mehdi Goudarzi:** Investigation. **Aliasghar Keramatinia:** Conceptualization. **Catherine WM. Ong:** Writing – original draft, Validation, Supervision, Methodology, Investigation, Conceptualization. **Delia Goletti:** Validation, Supervision, Conceptualization. **Lia D'Ambrosio:** Software, Investigation, Data curation. **Rosella Centis:** Methodology, Investigation. **Mohammad Javad Nasiri:** Writing – review & editing, Writing – original draft, Visualization, Validation, Supervision, Software, Resources, Project administration, Methodology, Investigation, Funding acquisition, Formal analysis, Data curation, Conceptualization. **Giovanni Battista Migliori:** Writing – original draft, Visualization, Validation, Supervision, Software, Resources, Project administration, Methodology, Investigation, Funding acquisition, Formal analysis, Data curation, Conceptualization.

## Ethics approval and consent to participate

Not applicable.

## Consent for publication

All authors have consented to the publication of this manuscript.

## Availability of data and material

All data and materials relevant to this study are included within the manuscript.

## Funding

This research was conducted with no external funding.

## Declaration of competing interest

The authors declare that they have no known competing financial interests or personal relationships that could have appeared to influence the work reported in this paper.
